# Deciphering the role of ectosomes in cancer development and progression: focus on the proteome

**DOI:** 10.1007/s10585-017-9844-z

**Published:** 2017-03-19

**Authors:** Magdalena Surman, Ewa Stępień, Dorota Hoja-Łukowicz, Małgorzata Przybyło

**Affiliations:** 1 Department of Glycoconjugate Biochemistry, Institute of Zoology, Jagiellonian University in Krakow, Krakow, Poland; 2 Department of Medical Physics, M. Smoluchowski Institute of Physics, Jagiellonian University in Krakow, Krakow, Poland

**Keywords:** Cancer, Ectosomes, Immunosuppression, Intracellular communication, Multidrug resistance, Tumor microenvironment

## Abstract

Ectosomes are small heterogeneous membrane vesicles generated by budding from the plasma membrane in a variety of cell types and, more frequently, in tumor cells. They are shed into the extracellular space and are proposed as a novel form of intracellular communication in which information is transmitted from the originating cell to recipient cells without direct cell-to-cell contact. This review focuses on a single population of extracellular vesicles—ectosomes. We summarize recent studies of tumor-derived ectosomes which examine their biogenesis and protein cargo, and their influence on different aspects of cancer progression. We discuss possible clinical implications involving ectosomes as potential biomarkers, diagnostic tools and treatment targets in oncology. The unique composition of the molecules (cargo) that ectosomes carry, and their functional role, depends largely on the state of their originating cell. Through horizontal transfer of a variety of biologically active molecules (including proteins, lipids and nucleic acids) between donor and recipient cells, tumor-derived ectosomes may play functional roles in oncogenic transformation, tumor progression, invasion, metastasis, angiogenesis promotion, escape from immune surveillance, and drug resistance, thereby facilitating disease progression. The presence of tumor-derived ectosomes in body fluids such as the blood and urine of cancer patients makes them potentially useful prognostic and predictive biomarkers. Tumor-derived ectosomes also offer possible targets for multiple therapeutic strategies.

## Introduction

Cancer development and progression are multistep processes in which a series of changes in the tumor microenvironment and in intercellular communication occur. Those highly specific alterations are driven mainly by endogenous molecular factors with proven oncogenic potential. Most bioactive molecules involved in carcinogenesis are directly secreted and act in an auto-, para- or endocrine manner, but another mechanism of their delivery to target cells has also been described. A variety of cell types, including cancer cells, are known to release extracellular vesicles (EVs)—small, membrane-enclosed particles which can mediate the transfer of different signaling factors, structural proteins, nucleic acids or lipids [1]. Following their in vivo release to the intercellular space, EVs typically are detected in a wide spectrum of body fluids such as blood (plasma or serum), urine, cerebrospinal fluid (CSF), bile, ascites, saliva, amniotic fluid, milk or semen; they can also be isolated in vitro from conditioned media of cultured cells. Such accessibility contributes to their prognostic, diagnostic and therapeutic value for particular health conditions [2].

Over the years, successive studies have revealed striking diversity within EV populations. Across this diversity they can be classified into several distinct populations based on their size, density, cellular origin, release mechanism and marker proteins [3]. In the EV-related nomenclature, one dominant naming convention of definitions has emerged recently [4–6], as outlined below.

**Fig. 1:**
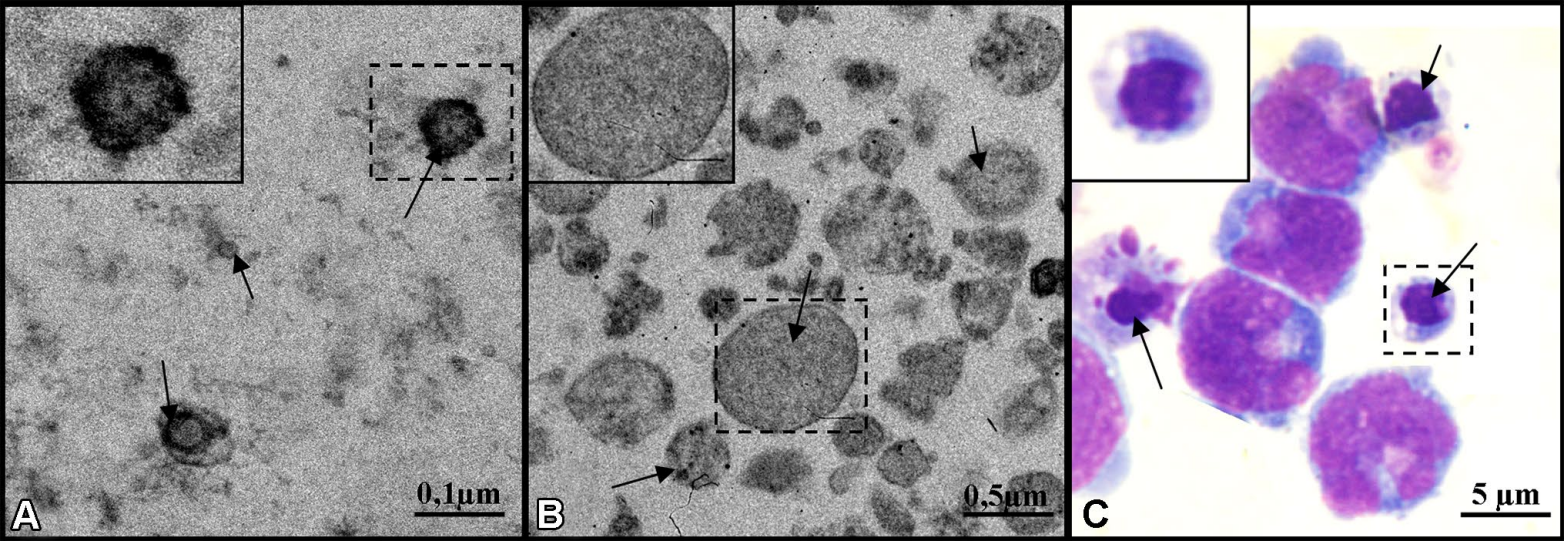
Representative images of extracellular vesicles released from tumor cells. **a** Exosomes from urine of diabetic patients (transmission electron microscopy, TEM), **b** ectosomes from human melanoma WM1205Lu cells (TEM), **c** apoptotic bodies from human acute lymphoblastic leukemia MOLT-4 cells (May-Grűnwald-Giemsa staining)

Ectosomes represent a fairly heterogeneous population of vesicles ranging in diameter from 0.1 to 1 µm (Fig. 1a). As a result of loss of calcium-dependent membrane phospholipid asymmetry and rearrangement of the cytoskeleton, ectosomes are formed by outward membrane budding and then are shed from the cell surface [1, 3, 7, 8]. Another population of smaller (30–100 nm) EVs called exosomes can be released by fusion of multivesicular bodies with the cell membrane, followed by exocytosis (Fig. 1b) [1, 3, 7, 8]. Finally, cells undergoing apoptosis and fragmentation also release vesicles formed by membrane protrusion. Unlike ectosomes, however, apoptotic bodies may contain cytosolic organelles or nuclear fragments, and they are considerably larger (up to 5 µm) (Fig. 1c) [3, 8]. Table 1 briefly characterizes these three populations of EV. Table 2 gives examples of molecular markers associated with ectosomes. Recently the existence of a new type of EVs termed sphereosomes has been postulated [9]. The presence of these structures, between 40 and 125 nm in size, was first described in gastrointestinal stromal tumor cells. Sphereosomes are believed to be formed through a newly found mechanism of shedding from multivesicular spheres.

**Table 1:** Comparison of three types of EVs: exosomes, ectosomes and apoptotic bodies

	Vesicle population		
	Exosomes	Ectosomes	Apoptotic bodies
Size in diameter	30–100 nm	100–1000 nm	1000–4000 nm
Appearance	Homogeneous	Heterogeneous	Heterogeneous
Sedimentation	100,000–120,000×*g*	16,000–20,000×*g*	5000–16,000×*g*
Filtration	20–200 nm	>200 nm	>1000 nm
Site of generation	Multivesicular bodies (MVBs)	Plasma membrane	Cells undergoing apoptosis
Type of generation	Constitutive	Regulated	Regulated
Mechanism of generation	Exocytosis of multivesicular bodies	Budding from plasma membrane	Cell shrinkage and death
Mechanism of sorting	Ceramide-dependent	Unknown	N/A
Intracellular storage	Yes	No	No
Main protein markers	Tetraspanins, HSP70, HSP90, Alix, Rab5a/b	β1 integrins, Selectins, CD40, MMP, Lineage markers, Ezrin	Histones
Lipid composition	Cholesterol, Ceramide	Phosphatidyserine, Cholesterol	Phosphatidyserine
Genetical information	Non coding RNA, micro RNA	mRNA, micro RNA	mRNA, micro RNA, DNA
Intact organelles	No	No	Yes
Anexin V binding	Poor	Strong	Strong
Impact on the immune system	Immunostimulators	Immunosuppressors	Immunosuppressors

**Table 2:** Molecular markers of ectosomes

Biomarker class	Name	Functions	References
Membrane associated proteins	Tissue factor (TF)	Thrombus formation, activation of cancer stem cells and angiogenesis	[10, 11]
	Integrins	β1 integrin—cell adhesion, CD41 (GPIIb/IIIa, αIIbβ3)—platelet aggregation and adhesion	[12, 13]
	ARF6	Remodeling of membrane lipids, regulation of ectosome release	[14, 15]
	Lineage markers	CD14 (monocytes), CD61 (platelets), CD62e (endothelium), CD66b (granulocytes), CD45 (leukocytes), CD4 (Th lymphocytes), CD8 (Ts lymphocytes), CD20 (B lymphocytes), Glycophorin A (erythrocytes)	[16]
	EGFR	Signal transduction	[17, 18]
	VAMP3	v-SNARE	[14]
	LAMP-1	Decreasing NK cells anti-tumor response	[19]
Membrane associated lipids	Flotillin-1	Lipid raft molecule	[20]
	Phosphatidylserine (PS)	Membrane phospholipid	[21]
	Sphingomyelin (SM)	Membrane phospholipid	[22]
Soluble	Proteases (MMP2, MMP9, uPA)	Degradation of extracellular matrix	[12, 23–26]
	CD147/basigin/EMMPRIN	Extracellular matrix metalloproteinases activator	[27]
	VEGF	Proangiogenic factor	[28]
	IL-1β	Inflammation cytokine	[29]
Cytoskeleton associated	Actin	Not defined	[30]
	Myosin	Pinching of vesicle neck during release of ectosome	[15]
	Ezrin	Multi drug-resistance	[31, 32]

**Fig. 2:**
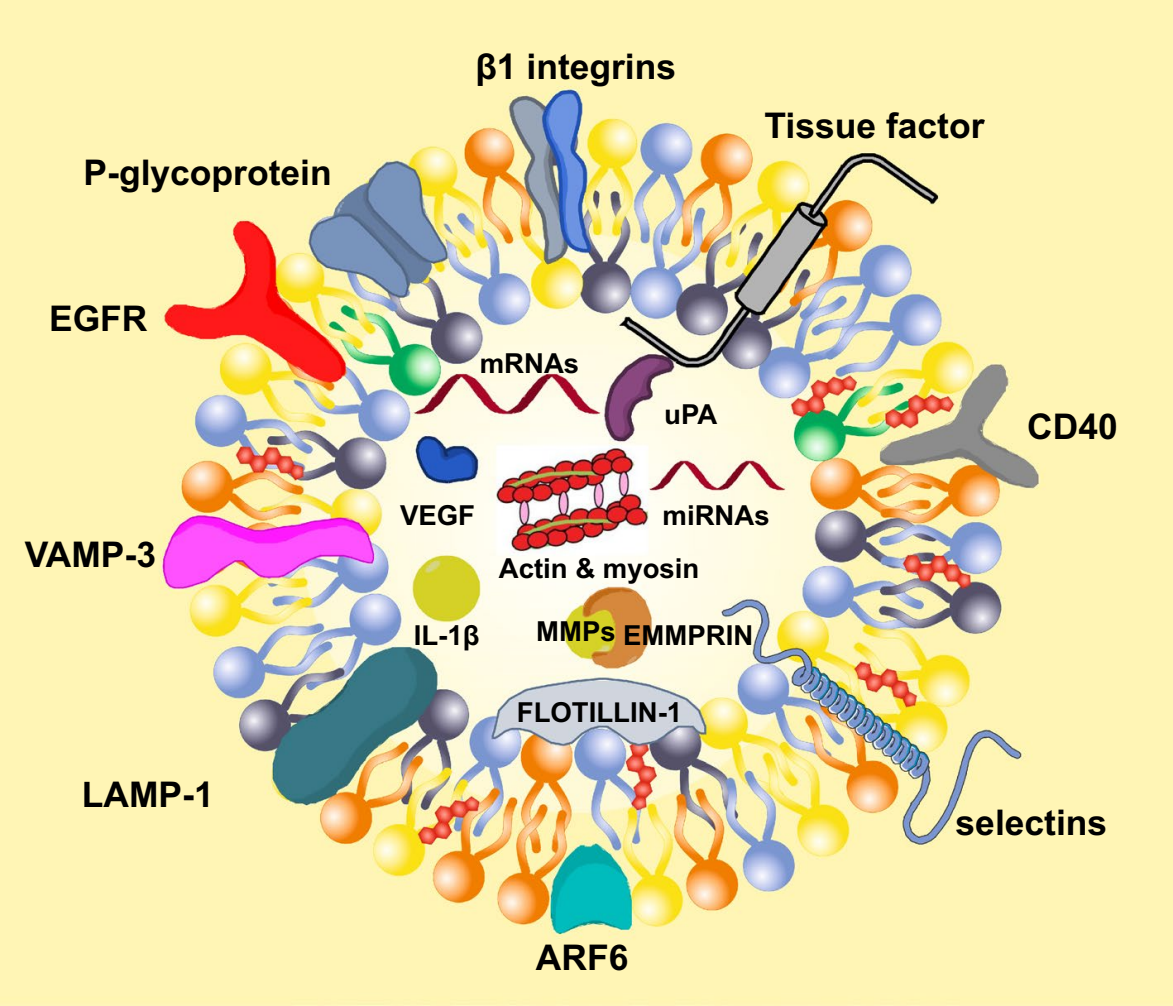
Examples of ectosome cargo. ARF6: ADP-ribosylation factor 6, CD40: cluster of differentiation 40, EGFR: epidermal growth factor receptor, EMMPRIN: extracellular matrix metalloproteinase inducer, IL-1β: interleukin 1β, LAMP-1: lysosomal-associated membrane protein 1, MMP: matrix metalloproteinase, uPA: urokinase plasminogen activator, VAMP-3: vesicle-associated membrane protein 3, VEGF: vascular epithelium growth factor

It is well established that EVs can be released under both physiological and pathological conditions. The particular function of an EV arises from the cargo it carries, which is highly dependent on the cell type from which a given EV originates [33]. Examples of ectosome cargo are shown in Fig. 2. The biological processes that may be controlled or modulated by vesicular content include coagulation, local inflammation, cell differentiation, vascular senescence and remodeling [4, 5, 7, 34, 35]. Cancer cells are known to release increased amounts of EVs, often described under a collective name: tumor-derived microvesicles (TMVs). The specific cargo that is horizontally transferred within a TMV affects a variety of cellular events during the respective stages of cancer progression. TMVs contain molecules directly stimulating invasion, metastasis and angiogenesis. Other components promote the acquisition of an aggressive phenotype or influence changes in the tumor microenvironment within the primary site and metastatic niche. TMVs may also facilitate transfer of the commonly used chemotherapeutics out of the cell, thereby contributing to multi-drug resistance. Finally, fusion of TMVs with immune cells often leads to inhibition or alteration of the immune response to cancer cells [1, 5, 36].

Our knowledge of the role of EVs in carcinogenesis is increasing vastly. This review focuses on a single population of EVs—ectosomes. We give the main points of recent studies of tumor-derived ectosomes which examine their biogenesis, proteome cargo, and the influence of that cargo on the different aspects of cancer progression. We discuss the clinical implications of the potential use of ectosome proteomes as biomarkers, diagnostic tools and treatment targets in oncology.

### Biogenesis of ectosomes

**Fig. 3:**
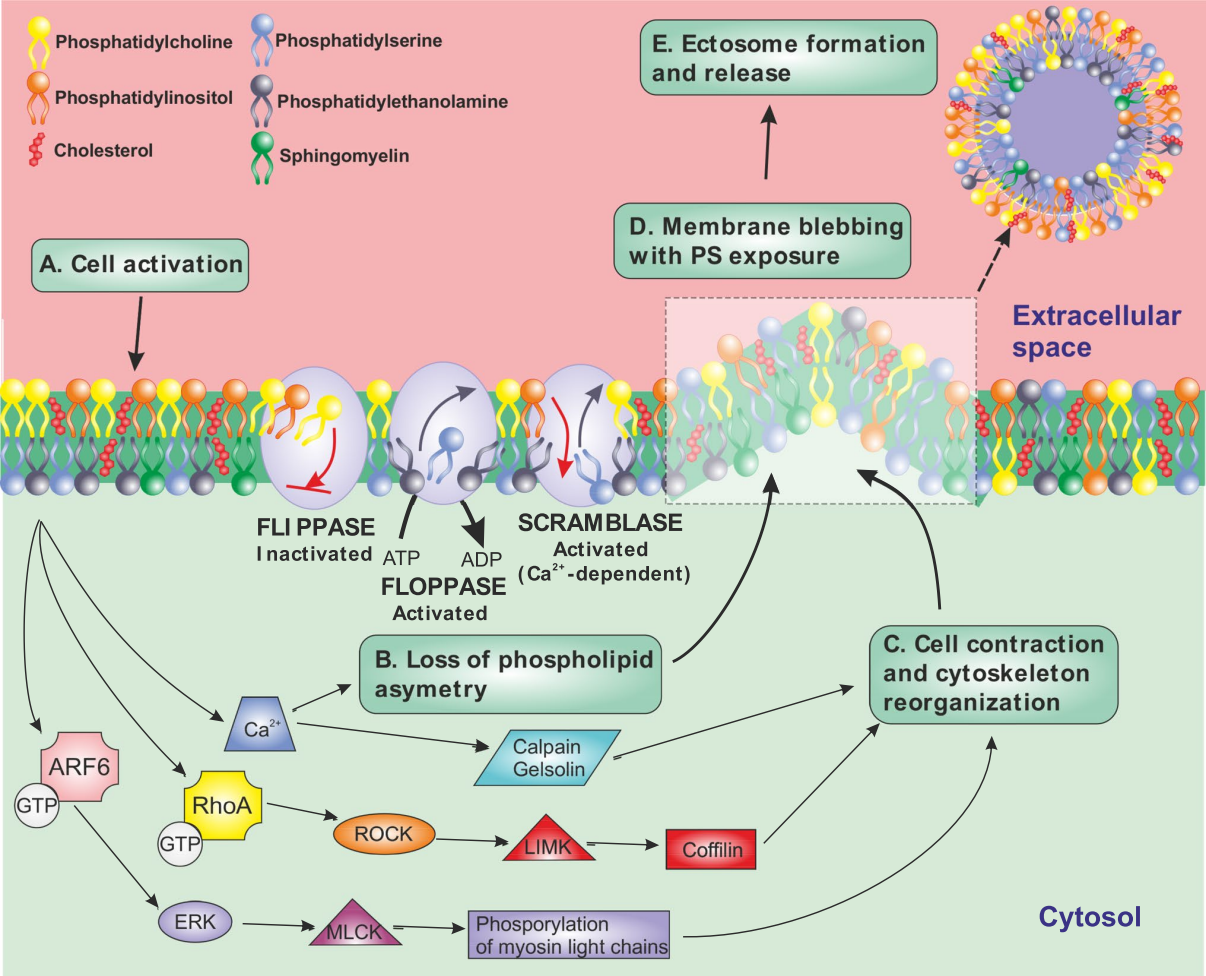
Mechanisms responsible for ectosome blebbing and release. Ectosomes are generated by outward budding and fusion of the plasma membrane, but their membrane composition is distinct from that of parental cells. Alterations of phospholipid symmetry are governed by aminophospholipid translocases (flippase and floppase) and Ca^2+^-dependent scramblase. Ectosomes are enriched in cholesterol, whereas phosphatidylserine (PS) is exposed on the extracellular leaflet of shed ectosomes. Cytoskeletal reorganization upon ectosome release is induced by calpain and gelsolin, although alternative mechanisms in cancer cells have been described, including RhoA [37] or ARF6 [14, 15] and their effectors. ARF6: ADP-ribosylation factor 6, ERK: extracellular signal-regulated kinase, LIMK: LIM domain kinase, MLCK: myosin light chain kinase, RhoA: Ras homolog gene family member A, ROCK: Rho-associated protein kinase

Better knowledge of EV biogenesis will be critical to an understanding of their role in intercellular communication, and eventually may allow this process to be regulated in different cell types, including cancer cells. Recent reports suggest that several types of EVs can be released from a single donor cell [29]. The formation of EVs entails the accumulation of their components in particular domains within the membranes of origin, which subsequently undergo budding (Fig. 3). While this initial assembly mechanism is similar in exosomes and ectosomes, the processes by which these two populations are released to the intercellular space differ significantly. In both cases the release of EVs is highly regulated and proceeds under the control of numerous molecular modulators [1, 7].

Since Wolf’s first description of ectosome-like particles originated from platelets [38], numerous studies have investigated the possible mechanisms of ectosome formation. Over the years it has become obvious that, unlike in exosomes, the release of ectosomes does not require exocytosis. It involves the formation of outward buds in specific regions of the cell membrane, followed by direct shedding and immediate release of the vesicle to the intercellular space [39]. These processes are initiated mainly via activation of a given cell by different agonists (e.g. ATP, growth factors, cytokines) and subsequently an increase in the level of intracellular Ca^2+^. Once additional Ca^2+^ is released from the endoplasmic reticulum, the cell membrane undergoes significant molecular rearrangement at the sites of ectosome origin, including changes in lipid and protein composition as well as in cytoskeleton structure [40].

Free calcium ions act as cytosolic secondary messengers, leading to the recruitment and activation/inhibition of several enzymes engaged in maintaining membrane asymmetry. Prior to ectosome release, phosphatidylserine (PS) and phosphatidylethanolamine (PE) are exposed on the outer leaflet of the cell membrane as a result of floppase and scramblase activation in response to strong Ca^2+^-mobilizing agents. At the same time, another translocase–flippase—is inhibited, thus preventing relocation of the aforementioned phospholipids [1, 3, 39]. A deficiency of Ca^2+^-dependent phospholipid scrambling activity, caused by mutation of the transmembrane protein anoctamin-6 gene (*ANO6*, also known as *TMEM16F*), reduces platelet activation and PS exposure on platelets and other cells, and it up-regulates a number of cytoskeleton, lysosome/peroxisome-related and phospholipid regulatory proteins [41]. PS-positive ectosomes can be distinguished easily from other circulating EVs with the use of flow cytometry by annexin V or lactadherin binding [21, 34, 42]. Increased Ca^2+^ levels may also facilitate degradation of the cytoskeleton structure and subsequent vesiculation by activating calcium-dependant proteases such as calpain and gelsolin [3]. Recent studies have identified RhoA, a member of the Ras protein superfamily of small GTPases, another regulator of ectosome release. By acting through its downstream effectors—a Rho-associated coiled-coil containing protein kinase (ROCK), LIM kinase (LIMK) and coffilin-RhoA altered the structure of actin–myosin filaments, leading to increased ectosome release by different cancer cell lines [37]. The release of ectosomes from invasive cells can be regulated by a GTP-binding protein, ARF6 [14, 15]. By activating extracellular signal-regulated kinases (ERK) and myosin light-chain kinase (MLCK), ARF6 regulates actin polymerization and phosphorylation of myosin light chains, leading to ectosome release [14].

## Biological role of ectosomes in carcinogenesis

The biological and clinical significance of ectosome secretion has been a subject of sustained research. The majority of evidence for the role of ectosomes in cancer is based on correlative studies in clinical and preclinical settings and on experiments done in vitro. In the first report confirming the release of ectosomes by cancer cells, from 1978, they were identified in cultures of spleen nodules and lymph nodes from a patient with Hodgkin lymphoma [43]. Soon thereafter, different melanoma cell lines were shown to release ectosomes [44, 45] which displayed the ability to boost metastatic potential when fused with less invasive tumor cells [44].

The particular effects exerted by ectosomes during cancer progression depend on their interactions with recipient cells. As described in a recent review, binding of ectosomes is most likely determined by different adhesion molecules, such as integrins [2]. Upon binding, ectosomes stimulate target cells by delivering surface receptors, or by directly inducing receptor-mediated signal transduction via transported ligands, or by delivering bioactive molecules (proteins, lipids, nucleic acids) after fusion with the cell membrane or after endocytosis [33, 46]. Regardless of the mechanism, an ectosomal cargo can modulate essential processes in associated cancer cells, as well as functions of fibroblasts [47], endothelial cells [48] and immune cells [49]. The described disruption of homeostasis within the tumor microenvironment, changes in the structure of the extracellular matrix (ECM) and transfer of oncogenic phenotypes all testify to the participation of ectosome release in different stages of cancer progression.

### Transfer of oncogenic phenotypes

Cancer development was long considered a consequence of multistep mutations of genetic material. Nowadays there is a shift from those strictly genocentric explanations for the transformation from normal to malignant cells, towards epigenetic and other non-genetic interpretations. Non-genetic mechanisms of phenotypic transformation may involve the transfer, via ectosomes, of membrane receptors for growth factors, RNA molecules or even lipids [50]. For instance, the oncogenic mutant of an epidermal growth factor receptor (EGFRvIII) was shown to be horizontally transferred within ectosomes released by glioma cells and taken up by a non-invasive population of tumor cells in vitro [17, 18]. Cells that acquired EGFRvIII exhibited activation of the MAPK and Akt signaling pathways, along with altered expression of genes regulating cell survival (BclxL) and proliferation (p27), resulting in a series of morphological changes and stimulation of anchorage-independent growth [17]. Ectosomes containing mRNA for EGFRvIII isolated from human glioblastoma sections consistently stimulated in vitro proliferation of malignant glioma cells (U87) [48].

Other findings highlight the role of ectosomes in the transformation of normal cells within the tumor microenvironment. Antonyak et al. [47] demonstrated that ectosomes released by breast cancer and glioblastoma cell lines (MDAMB231 and U87) contain tissue transglutaminase (tTG) and its tTG-binding partner and cross-linking substrate, fibronectin (FN). Once transferred to the recipient fibroblasts via ectosomes, tTG and FN acted cooperatively to induce their transformation by activating mitogenic signaling (phosphorylation of FAK and ERK kinases), leading to increased cell survival and aberrant growth [47].

### Tumor cell invasion and metastasis

The metastatic cascade begins with local invasion of primary tumor cells within the surrounding tissue. Once the cells pass the barrier of the basement membrane, they are able to penetrate the lumen of the blood vessels (intravasation) and then travel with the blood stream to predetermined metastatic sites. Subsequent migration of tumor cells from the blood to the target tissue (extravasation) starts the process of secondary tumor growth. The results of numerous studies support the suggestion that various molecules of the ectosomal cargo play a significant role at each stage of the metastatic cascade [36].

The invasive and migratory properties of tumor cells are highly dependent on the activity of different proteases such as matrix metalloproteinases (MMPs). By degrading particular components of the ECM, this group of enzymes promotes the mobility of migrating cells and creates a path of less resistance. MMPs are also responsible for proteolysis within the basement membrane, which allows tumor cells to penetrate the lumen of blood and lymphatic vessels and metastasize [36]. The presence as well as the proteolytic activity of MMP-2 and MMP-9 have been identified in ectosomes released in vitro by fibrosarcoma (HT1080) [51], ovarian (CABA1, A2780) [23, 28], breast (8701-BC) [12], prostate (PC3, LnCaP) [24, 25] and lung (HTB 177, CCL 185) [26] cancer cell lines.

It is well established that the activity of MMPs is regulated at several levels, including transcription, enzyme activation/inhibition, complex formation, and compartmentalization [52]. Supporting this, studies using fibrosarcoma and prostate cancer cell lines showed that urokinase plasminogen activator (uPA) is associated with ectosomes released in vitro. Addition of plasminogen to the ectosomal fraction resulted in activation of zymogens, indicating a role of the urokinase-plasmin system in MMP-2 and MMP-9 activation [24, 25, 51]. The activity of vesicle-associated proteases may also be influenced by the pH of the tumor microenvironment. In solid tumors the extracellular pH is acidic due to elevated anaerobic glycolysis and impaired clearance of metabolic waste products. It has been found that low pH may promote the invasiveness of ectosomes by cathepsin B-mediated activation of gelatinases. Exposing vesicles released by ovarian cancer cells (CABA1) to acidic medium increased MMP-2 and MMP-9 activities; this effect was abolished by the specific inhibitor of cystein protease or by silenced expression of cathepsin B in CABA1 cells [53]. CD147/extracellular MMP inducer (EMMPRIN), a membrane glycoprotein, may also be involved in the progression of malignancies via regulation of the expression of MMPs in tumor cells. CD147/EMMPRIN-bearing ectosomes derived from breast cancer cell lines (MCF-7, SK-BR-3, MDA-MB231) induced invasion of both autologous and heterologous cells. This effect was not mediated by matrix metalloproteinases, which were absent in the released ectosomes, but rather by activation of the p38/MAPK signaling pathway in tumor cells [27].

Proteolysis within the ECM can also result from the activity of adamalysin metalloproteinases with disintegrin and thrombospondin domains (ADAMTSs). Ectosomes shed by oligodendroglioma cells exhibited aggrecanase activity in vitro, cleaving aggrecan at sites previously identified as targets for different ADAMTSs: ADAMTS1, ADAMTS4 and ADAMTS5. Immunodetection of the cleaved fragments showed one or more of these enzymes to be responsible for the ectosome activity [54].

Ectosomes may promote cancer invasion and metastasis indirectly by altering normal cell function. Through interactions with cancer ectosomes, stromal cells such as fibroblasts contribute to the creation of a favorable niche for cancer development. In studies by Castellana et al. [24], fibroblasts were activated in vitro by ectosomes derived from a highly metastatic prostate cancer cell line (P3C). After incubation, the fibroblasts exhibited ERK phosphorylation and up-regulation of MMP-9. Moreover, the activated fibroblasts themselves released vesicles which in turn were able to boost the invasiveness and migration of P3C cells. A similar observation was reported in yet another study [55] confirming the role of tumor-derived ectosomes in activating stromal cells within the tumor microenvironment in vivo and in vitro. Ectosomes released by cancer stem cell populations (isolated from renal carcinoma cells) activated mesenchymal stromal cells (MCSs) and up-regulated the expression of MMP-1 and MMP-3 in MSCs. Mice inoculated with renal carcinoma cells (K1) previously cultured with activated MSCs showed increased tumor growth versus the control group. Finally, ectosomes released in vitro by MDA-MB-231 carcinoma cells enhanced collagenase activity in fibroblasts (MCF10a cell line). In that case, reorganization of 3D collagen matrices by ectosome-stimulated fibroblasts was associated with increased FAK phosphorylation [56].

Matrix degradation and subsequent tumor invasion have also been correlated with elevated expression of urokinase and other components of the plasminogen activation system. Schroder et al. [57] recently demonstrated that SerpinB2 (plasminogen activator inhibitor type 2/PAI2) is expressed on the surface of ectosomes released by B16 melanoma cells. SerpinB2-expressing ectosomes significantly reduced urokinase (uPA) activity in vitro as compared with vesicles obtained from control cells that did not produce SerpinB2. This mechanism was later used to explain the decreased migratory properties of SerpinB2-expressing B16 cells in Transwell assays, suggesting that ectosome-associated SerpinB2 may also inhibit uPA-mediated cancer invasion, migration and metastasis in vivo [57].

### Angiogenesis

In the course of carcinogenesis, enlargement of the tumor requires expansion of the vascular network. Neovascularization allows effective delivery of substances essential for survival of tumors and facilitates their subsequent migration to metastatic sites. Initiation of angiogenesis in tumor lesions is associated primarily with activation of various signaling pathways in tumor cells, leading to proliferation and migration of vascular endothelial cells or their precursors.

Ectosomes released by different cancer cells have been shown to facilitate the transfer of several proangiogenic factors or to up-regulate their expression in endothelial cells. Studies by Taraboletti et al. [28] demonstrated that ectosomes isolated from two ovarian cancer cell lines (CABA1, A2780) contained significant amounts of matrix-degrading metalloproteinases (MMP-2, MMP-9) and vascular epithelium growth factor (VEGF), and that these factors stimulated the motility and invasion of endothelial cells into Matrigel. In other work, human umbilical cord vein cells (HUVECs) showed up-regulated expression of autocrine VEGF upon uptake of EGFRvIII from ectosomes released by two human epithelial carcinoma cell lines (A431, A549) [18]. In another study, induction of the angiogenic phenotype in HUVECs was attributed to the presence of CD147/EMMPRIN in ectosomes derived from three ovarian cancer cell lines (OVCAR3, SKOV3, A2780). CD147/EMMPRIN-positive vesicles stimulated in vitro proliferation, invasiveness and expression of MMP-2 and MMP-9 in HUVECs [27]. Another proangiogenic factor is IL-6, whose production (along with VEGF) by the EA.hy926 endothelial cell line was increased upon incubation with ectosomes derived from human multiple myeloma cells. Ectosomes from myeloma cells induced proliferation and invasion of EA.hy926 cells, as determined in Transwell cell invasion assays [58].

The crucial role of ectosomes in tumor angiogenesis has also been confirmed in animal studies. Munster et al. [59] obtained two distinct populations of ectosomes from EMT/6 breast cancer cells exposed or not exposed to anti-VEGF antibody (B20). As expected, mice inoculated with ectosomes released by B20-untreated cells showed higher mobilization of endothelial precursor cells and their colonization in growing tumors, as well as increased microvessel density [59]. These data suggest that cancer-cell-derived ectosomes stimulate the paracrine mechanism of endothelial cell proliferation in both a VEGF-dependent and a VEGF-independent manner. However, the most important role in cancer neovascularization may well be recognized in the endothelial-to-mesenchymal transition (EMT), a newly identified type of cellular transdifferentiation responsible for vascular system development and repair [60, 61].

At present, much less is known about the role of membrane lipids transported within ectosomes in tumor angiogenesis. Kim et al. [22] identified sphingomyelin in ectosomes shed from HT1080 fibrosarcoma cells as the active component for ectosome-induced endothelial cell migration, in vitro tube formation (Matrigel tube formation assay), and *ex ovo* neovascularization of the chick chorioallantoic membrane; comparable effects on endothelial cell migration and angiogenesis were exerted by lipid extracts from ectosomes and purified sphingomyelin, but were not observed in the case of lipid extracts previously treated with sphingomyelinase [22]. Besides growth factors, metalloproteinases, cytokines and lipids, ectosomes may supply endothelial cells with proangiogenic miRNAs. Transfer of pro- and anti-angiogenic miRNA from cancer to endothelial cells via ectosomes may promote the formation of blood vessels by altering the translation of particular proangiogenic factors, or it may cause down-regulation of VEGF expression in a microRNA-specific manner [47, 62]. Among the different microRNA and protein cargos identified in human collateral cancer ectosomes, miR-1246 and TGF-β have been demonstrated to exert their pro-angiogenic effects by activating Smad 1/5/8 signaling in HUVECs [63].

Another important example of pro-angiogenic cancer ectosomes and cell interactions is the contribution of platelet-derived microvesicles (PMVs) in carcinogenesis and neovascularization [6]. Below we cover what is currently known about this.

### Cancer-induced thrombosis

Since cancer progression is often associated with increased platelet activation and aggregation, PMVs are thought to be mediators in platelet-tumor interactions. Tumor cells activate the production of thrombin, a common agonist of platelets, which induces ectosome shedding [64]. For example, the supernatant obtained from a human neuroblastoma cell line (NCG) induced platelet aggregation via thrombin-induced procoagulant activity [65]. CD41 (GPIIb/IIIa, αIIbβ3) and P-selectin are specific antigens for activated platelets. Their presence on the surface of ectosomes promotes adhesion of cancer cells to the vascular endothelium and facilitates their extravasation [11]. Adhesion of platelets and circulating cancer cells is regulated mostly by the ligand-receptor mechanism of PSGL-1/P-selectin interaction, and the presence of P-selectin on the surface of PMPs may facilitate binding of P-selectin-positive ectosomes to PSGL-1-expressing cancer cells and thereby increase cancer invasiveness [11, 66]. P-selectin- and PSGL-1-dependent accumulation of circulating PMVs in vascular injury foci has been described as an important mechanism of ectosome delivery to thrombi and of tissue-factor-dependent fibrin generation [67].

Among the numerous specific procoagulant molecules, tissue factor (TF) is the major initiator of thrombin activation in blood coagulation pathways. A widely discussed question is whether PMVs contain platelet-originated TF, or if this activator is incorporated into PMVs due to binding of TF-positive EVs derived from extravascular cells and macrophages to PMVs or platelets, or if TF is de novo expressed in activated platelets [68]. It is now commonly accepted that two forms of TF are present in the circulatory system: full length (flTF) and alternatively spliced (asTF) [69]. The extracellular domain of flTF was found to initiate coagulation by binding coagulation factor VII or its activated form (VIIa) to make a membrane-bound complex which activates coagulation factor X. Oncogenic transformation caused by the *RAS* mutation and loss of *p53* resulted in TF up-regulation [70]. Later it was shown that ectosome-mediated transfer of TF between two breast cancer cell lines changed cell TF expression related to their aggressiveness potential [71]. Therefore it is highly likely that PMVs contribute to the transfer of TF-positive ectosomes from macrophages and different populations of cancer cells, and that they can facilitate the propagation of TF-related aggressive phenotypes [11, 16, 71, 72]. TF-bearing microvesicles arise from lipid rafts and then fuse with activated platelets through a PSGL-1-dependent mechanism; their shedding was significantly reduced under conditions of depleted membrane cholesterol [20, 67]. The molecular mechanism for activation of TF and other coagulation factors also involves PS exposure. Presentation of negatively charged PS on the surface of ectosomes is closely related to exposure of binding sites for coagulation factors Va, VIII and X, which leads to their activation and phosphatidylserine-dependent initiation of coagulation pathways [11].

In contrast to the procoagulatory activity of ectosomes, anticoagulation and antimetastatic effects of tumor-derived large microvesicles have been reported. Expression of plasminogen activator inhibitor type 2/PAI-2 (SERPINB2) effectively inhibits urokinase activity, and was associated with favorable prognoses [57]. Work by Mezouar et al. [73] showed that inhibition of platelet activation with clopidogrel (an anti-platelet agent) in an in vivo mouse model prevents P-selectin- and (αvβ1, αvβ3) integrin-mediated accumulation of ectosomes at the site of thrombosis. Upon treatment with clopidogrel, animals bearing pancreatic tumors showed a decrease of tumor growth and metastasis. Taken together, these observations indicate that the use of anti-platelet drugs may improve the efficacy of anticancer therapy and slow the rate of disease progression.

### Influence on the immune system

The immune system is well adapted to impede cancer progression, although its role remains inessential until the accumulated genetic changes are fixed. At that point, spontaneous cancer immunity may arise to contain tumor growth in early stages of its progression; however, many types of cancer cells have developed a number of mechanisms allowing them to evade immune surveillance. Ectosomes and other MVs are widely associated with suppression of the immune response to transformed cells, and several theories explaining their role have been proposed [36, 74–76].

Numerous studies indicate that tumor-derived ectosomes may induce chemotaxis of blood leukocytes. In vitro, ectosomes released by pancreatic adenocarcinoma (HPC-4), colorectal adenocarcinoma (DeTa) and lung carcinoma (A549) cell lines stimulated the chemotactic activity of granulocytes, lymphocytes and monocytes. Those results were obtained using Transwell chambers and were attributed to several chemokines (particularly IL-8) transferred inside ectosomes [74]. Upon interaction with immune cells, ectosomes can interfere with the T-cell response by altering the differentiation of antigen-presenting cells, or else can inhibit functions of effector cells [36]. For example, Köppler et al. [75] described the immunosuppressive properties of ectosomes released in vitro by the Kato gastric carcinoma cell line. Incubation with isolated vesicles interfered with the activation of monocytes by lipopolysaccharide (LPS), resulting in decreased release of tumor necrosis factor α (TNF-α) and granulocyte macrophage colony-stimulating factor (GM–CSF). Another mechanism was shown for ectosomes from melanoma and colon carcinoma cell lines [76] that inhibited the differentiation of monocytes to antigen-presenting dendritic cells. Moreover, the remaining population of monocytes released an immunosuppressive cytokine—transforming growth factor β (TGF-β)—which inhibited T-cell cytolytic activity.

The immunosuppressive effects exerted by ectosomes do not appear to be constant and universal. Vesicles isolated from pancreatic adenocarcinoma (HPC-4), colorectal adenocarcinoma (DeTa) and lung carcinoma (A549) cell lines induced the production of proinflammatory cytokines (TNF-α and IL-12) and reactive oxygen intermediates (ROIs) by monocytes [77]. As a result, stimulated monocytes showed significantly increased cytotoxic/cytostatic effects on cancer cells in vitro, as compared with control monocytes. Despite the enhancement of the antitumor response, increased production of the anti-inflammatory cytokine IL-10 was also observed [77]. An explanation for such a discrepancy has recently been suggested by another study. Ectosomes released by colon cancer cell lines (Caco-2, SW480, SW620, LoVo) were shown to influence the differentiation of monocytes to macrophages, resulting in their variable polarization status (M1/M2, proinflammatory/anti-inflammatory) [78]. The impact of ectosomes on macrophage differentiation and cytokine production depended on the timing of the monocytes’ contact with isolated vesicles. Immediate exposure resulted in the highest release of IL-10, whereas monocytes incubated with ectosomes on the sixth day of culture exhibited the strongest secretion of IL-12 and TNF-α (corresponding to the increased fraction of proinflammatory M1 monocyte-derived macrophages). Macrophages that differentiated after prolonged exposure to vesicles (days 0, 3 and 6) secreted the lowest amounts of IL-12 and TNF-α, probably due to deactivation of monocytes/macrophages by ectosomal hyaluronan fragments [78, 79]. Observations made in the last-mentioned time regime most likely reflect the immunosuppressive effect of prolonged ectosome exposure during cancer progression in vivo.

Interactions of tumor-derived ectosomes with immune cells may also indirectly regulate different aspects of cancer progression, including angiogenesis. In studies by Baj-Krzyworzeka et al. [74], ectosomes released by pancreatic adenocarcinoma (HPC-4), colorectal adenocarcinoma (DeTa) and lung carcinoma (A549) cell lines increased in vitro secretion of proangiogenic cytokine IL-8 by monocytes. Later the proangiogenic potential of ectosome-treated monocytes was tested in vivo in a mouse model. Matrigel matrixes containing stimulated or not stimulated monocytes were implanted into NOD-SCID mice and excised 7 days later. Hemoglobin content in excised Matrigel matrixes showed stronger proangiogenic activity of monocytes previously incubated with ectosomes released by cancer cells [74].

Finally, it is important to note that the majority of comprehensive studies have focused on the exosomal fraction of microvesicles, so the data on the effects of ectosomes on the immune system are still very limited. It is very likely that effects attributed to exosomes, such as induction of effector cell apoptosis, stimulation of suppressor cell differentiation, or loss of antigens essential for recognition by NK cells and cytotoxic T-cells [80, 81], can also be exerted by ectosomes. Additional studies are needed to shed light on this question.

### The role of ectosomes of non-cancer origin

As described in previous sections, ectosomes released by cancer cells exert multiple regulatory effects during different stages of cancer progression. An increasing number of studies also implicate vesicles derived from non-transformed cells (such as fibroblasts and immune cells) or platelets in disease development. For instance, ectosomes released by immune cells (isolated from mouse spleen) induced migration of hepatocarcinoma (H22) and melanoma (B16) cell lines in vitro and hepatic cancer metastasis in mouse in vivo. These observations were related to the transfer of integrin αMβ2 (CD11b/CD18) from immune cells to ectosomes and then to tumor cells. The use of antibodies against either CD11b or CD18 led to significant decreases in ectosome-mediated tumor cell migration and metastasis [82]. In other work, ectosomes released in vitro by cancer-associated fibroblasts increased the proliferation of pancreatic cancer cells (DU145 cell line). Moreover, the utilization of lactate in anabolic processes was higher when DU145 cells received fibroblast proteins via ectosomes in Transwell or coculture conditions, suggesting that the acquisition of enzymes of the second step of glycolysis (subsequently identified in isolated ectosomes) may contribute to the metabolic shift of DU145 cells towards a reverse Warburg phenotype, more efficient in highly proliferative conditions [83].

Other reports support the suggestion that ectosomes released by platelets and megakaryocytes also contribute to cancer progression. They are constitutively produced in physiological conditions, though elevated levels of them have been observed in patients with various types of cancer, frequently correlated with disease stage, the presence of metastases, or survival [11]. In vivo and in vitro studies confirmed the involvement of platelet-derived vesicles (PMVs) in tumor growth, invasion and angiogenesis through interactions with cancer or endothelial cells. Janowska-Wieczorek et al. [10] showed that incubation with platelet-derived ectosomes resulted in higher expression of mRNA for several proangiogenic factors (MMP-9, VEGF, IL-8, HGF) by different lung cancer cell lines, and their increased adhesion to endothelial cells in vitro. Up-regulation of MMP-2 expression and activation of selected proliferative signaling pathways was also observed in cells cultured in the presence of vesicles. After injection into mice, cells previously incubated with ectosomes induced more lung cancer metastatic foci than control cancer cells [10]. A complete characterization of circulating EVs in colorectal cancer patients revealed that microvesicles obtained by 15,000×*g* centrifugation (ectosomes) are mostly CD41- and CD61-positive (platelet origin) and may act as conveyors of cancer-derived smaller vesicles [13].

## Potential clinical applications

The presence of ectosomes in various body fluids points to their potential use as biomarkers or prognostic indicators of cancer development and progression. Tumor-specific markers exposed on the surface of ectosomes might serve as confirmatory tools during the diagnostic process, whereas specific changes in the number of released vesicles or in their molecular composition appear to be highly indicative of disease stage and treatment efficacy.

Ectosomes are also being studied for possible improvement of clinical outcomes, mainly with regard to inhibition of the vesiculation process or for drug delivery; both strategies may benefit cancer management [8]. The clinical applications of ectosomes are still in development; their full potential is yet to be realized.

### Diagnostic value

Ectosomes generally contribute to the pathogenesis of cancer, but some of their unique characteristics can be exploited as diagnostic, prognostic and surveillance indicators for cancer patients. Elevated levels of ectosomes as compared with those of healthy controls have been detected in peripheral blood samples from patients with glioblastoma [84], non-small-cell lung carcinoma [85] and multiple myeloma [86]. On the other hand, ectosome levels were found to be significantly lower in colorectal carcinoma patients; thus, increased vesiculation may not be a universal characteristic of all types of cancer. The variation of ectosome release levels has been used to distinguish benign tumors from malignant breast [87] and prostate [88] cancers. Such differences were not observed between benign colorectal disease and colorectal carcinoma [89]. Increased plasma levels of ectosome-bearing tissue factor may indicate a highly invasive and poorly differentiated type of pancreatic cancer more able to infiltrate peripancreatic vessels [90].

A thorough determination of ectosomal molecular status may allow detection of specific cancer biomarkers. Ectosomes obtained from patients with malignant breast cancer exhibited elevated expression of several surface antigens (CD66; human epidermal growth factor receptor 2, Her2/neu; breast cancer resistance protein, BRCP; Hsp27) as compared with benign tumors, suggesting their potential use as relevant diagnostic markers for malignancy [87]. In other work, ectosomes released during progression of colorectal and pancreatic cancers were found to express surface glycoproteins such as mucine1 (MUC1), carcinoembryonic antigen (CEA) and carbohydrate antigen 19-9 (CA19-9). The number of MUC1- and CA19-9-positive vesicles differed significantly between the two cancer types, with MUC1 expression higher in colorectal cancer and CA19-9 expression higher in pancreatic cancer. Since MUC1 and CA19-9 are already being used in histopathology as differential markers for digestive system cancers, isolation of ectosomes from peripheral blood may obviate the need for invasive biopsy procedures in the future [88]. Ectosomes found in the blood could also furnish a novel prognostic tool to monitor malignant cells in multiple myeloma, where elevated numbers of CD138-positive vesicles have been correlated with the tumor burden [86].

The diagnostic and prognostic uses of elevated numbers of ectosomes, or of the presence of vesicles bearing certain molecules, depend on the establishment of proper isolation protocols. To gain valid information for clinical practice, optimal concentrations of uncontaminated vesicle populations are required. The several methods of obtaining such samples are based on vesicle size or density, or on marker expression. In general, ectosomes can be selected by differential centrifugation, immunoaffinity isolation (adsorption to magnetic/non-magnetic microbeads) or size exclusion chromatography [91, 92]. Unfortunately, so far no single isolation protocol can guarantee complete recovery of ectosomes from samples, nor ensure maintenance of their native form and function.

### Management of multidrug resistance

A number of reports suggest that ectosomes are among the critical factors in multidrug resistance (MDR), which remains an obstacle in cancer chemotherapy. MDR is associated with several mechanisms responsible for compromising the effectiveness of different chemotherapeutics. The mechanisms include changes in the rate of drug uptake and efflux, altered drug metabolism, decreased drug-target complex formation, and enhanced DNA repair [93]. MDR cancer cells have already been shown to overexpress different transporter proteins involved in the efflux of anticancer drugs including P-glycoprotein (Pgp), multidrug resistance-associated protein 1 (MRP1) and breast cancer resistance protein (BCRP) [94]. Recent in vitro studies demonstrated transference of functional Pgp [95, 96] or MRP1 [97] and the mRNAs for both proteins [97] via ectosomes released by MDR chronic/acute myeloid leukemia cells to drug-sensitive cells which subsequently acquired MDR phenotypes. Some of these transporter proteins are transferred alongside CD44, ERM (ezrin, radixin, moesin) protein family and cytoskeleton proteins within the ectosomal cargo [31]. Ezrin is known to determine Pgp membrane localization through cytoskeletal association, as shown in leukemic and breast cancer cells [31, 32]. de Souza et al. [96] found that upon incubation with ectosomes bearing different inhibitors of apoptosis proteins (IAPs), drug-sensitive human breast adenocarcinoma (MCF7) and human lung carcinoma (A595) cells were more resistant to apoptosis when treated with cisplatin or paclitaxel.

Apart from delivering transporter proteins, ectosomes may also directly facilitate the expulsion of chemotherapeutics from tumor cells and promote their survival. Doxorubicin-treated MCF7 human breast adenocarcinoma cells accumulated and released the drug in shed microvesicles [98]. This observation implied that modulatory interventions in the vesiculation process may offer a solution for various MDR cases, so studies were carried out later to verify that suggestion. Jorfi et al. [93]. described in vitro sensitization of pancreatic cancer cells (PC3 cell line) to docetaxel upon treatment with vesiculation-inhibiting calpeptin (calpain inhibitor). As a result, 20-fold lower concentrations of docetaxel used in the presence of calpeptin induced the same degree of apoptosis in PC3 cells as docetaxel alone. Inhibition of ectosome budding similarly improved the effectiveness of a combination chemotherapy (docetaxel and methotrexate) and also reduced the docetaxel dose required to limit tumor growth in mouse in vivo. Another study pointed to the therapeutic potential of peptidylarginine deiminases (PADs), a family of enzymes responsible for post-translational conversion of protein-bound arginine to citrulline [99]. PADs have been associated with deimination of cellular actin, which in turn rearranges the actin cytoskeleton and may facilitate vesiculation. Treatment of PC3 cells with chloramidine (PAD inhibitor) significantly reduced ectosome release and increased the sensitivity of PC3 cells to the cytotoxic effect of methotrexate.

In contrast to the presented findings, clinical outcomes may be improved by stimulation of the vesiculation process in certain types of cancer. Different ectosome-releasing agents are considered as potential alternative drugs in, for example, differentiation therapy against acute myeloid leukemia. Anso-Adda et al. [100] found that promonocytic leukemia cells (THA-1) released increased amounts of ectosomes upon stimulation with phorbol myristate acetate, all-trans retinoic acid and histamine. Isolated vesicles containing TGF-β1 inhibited the proliferation of THA-1 cells, and they induced differentiation of those cells to monocytes/macrophages.

### Therapeutic use

Ectosomes can carry a multitude of bioactive molecules. Potentially they offer a unique carrier system to deliver different therapeutic agents to cancer cells. Obvious advantages include easy preparation and manipulation, no restriction regarding the physicochemical properties of drugs, and the lack of autoimmune reaction due to the autochthonous origin of isolated vesicles [101]. Since ectosomes carry a variety of cancer-related surface receptors and adhesion molecules, they might be easily transported to specific tumor sites, enabling local drug delivery.

Recent preclinical and clinical trials have already exploited synthetic liposomes to successfully deliver chemotherapeutics such as doxorubicin or daunorubicin to in vitro cultures of melanoma cells [102] or in leukemic patients [103, 104]. The observed anticancer effects (decreased tumor growth [101] or prolonged survival after leukemia relapse [103, 104]) suggest that refinement and modification of natural vesiculation processes may allow ectosomes to be used as novel therapeutic vehicles. Tang et al. [101] demonstrated that malignant hepatocarcinoma cells (H22 cell line) incubated with doxorubicin, cisplatin or methotrexate subsequently released drug-containing ectosomes. Isolated vesicles had a cytotoxic effect on tumor cells in vitro and reduced hepatocarcinoma and ovarian cancer growth in mouse in vivo. Moreover, the ectosome-encapsulated chemotherapeutic agents showed higher efficacy and less adverse effects than drugs administered directly.

Ectosomes can also be used as vaccines in cancer management. In studies by Zhang et al. [105], 50% of mice immunized with ectosomes isolated from hepatocarcinoma (H22), melanoma (B16) and colon carcinoma (CT26) cell lines remained tumor-free after injection of cancer cells. Vesicles from the vaccines were taken up by dendritic cells and induced the expression of type-I IFN by activating the pathway of the cyclic GMP-AMP synthase/stimulator of interferon genes (cGAS/STING). Type-I IFN enhanced the maturation of dendritic cells that activated tumor-specific T-cells, leading to cytolysis of cancer cells [105, 106]. Only 12.5% of mice immunized with exosomes released by the same cell lines did not develop tumors, suggesting that ectosomes are more immunogenic and are a better option for developing cancer vaccines [105].

Ran et al. [107] demonstrated that ectosomes can also deliver an oncolytic adenovirus into the nucleus of tumorogenic cells and thus are potential agents for virotherapy. The applied vesicles were fatal to tumor cells cultured in vitro and also reduced tumor growth in vivo in adenocarcinoma mice. Moreover, the cytolytic activity of virus-containing ectosomes was more efficient than that of a free virus, because they appeared to be resistant to virus antibodies. Finally, ectosomes have also been shown to transfer miRNAs that regulate target gene expression and functions of recipient cells [108, 109]. The use of vesicles containing particular miRNAs in gene therapy clearly holds great promise, but so far no studies involving different models of cancer disease have been carried out.

## Concluding remarks

**Fig. 4:**
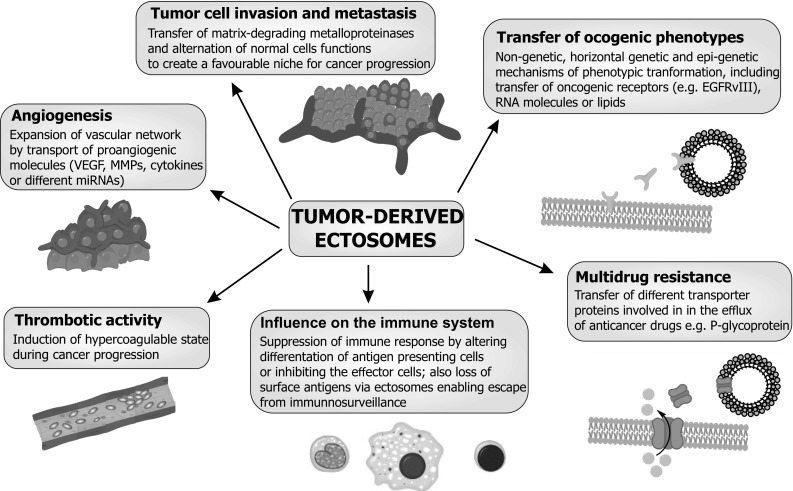
Role of tumor ectosomes in key pathways promoting cancer progression through interaction with local and distant cells

In this review we summarized the recent literature on the properties and biogenesis of tumor-derived ectosomes, as well as their potential roles in cancer growth. Revealing their biological roles is only the beginning. This branch of research has entered a phase of rapid progress. Tumor-derived ectosomes are now recognized to play significant roles in tumor development, facilitating the spread and release of cancer cells to generate metastases. Work on ectosomes has shed new light on the pathogenesis of malignant disease. They are a reservoir of biological information and a vital component of the specific microenvironment of the cell to the tumor microenvironment and to recipient cells, tumor-derived ectosomes provide a unique means of cellular export and cell-to-cell transport of insoluble bioactive molecules such as nucleic acids, membrane receptors and signaling molecules which affect recipient cell metabolism, mRNA processing, cell growth and motility, as well as angiogenesis and immune system functioning (Fig. 4). Elevated levels of tumor-derived ectosomes are associated with a variety of cancers, including brain [84], lung [85], breast [87], prostate [88], pancreatic [90] and gastric [108] cancers, as well as acute promyelocytic leukemia [110]. As it is well established that the number of tumor-derived ectosomes increases with cell invasiveness or disease progression [14], potentially these vesicles can serve as prognostic biomarkers of disease stages and of treatment efficacy, and can be effective targets for anticancer therapies.

## Abbreviations

ADAMT: Adamalysin metalloproteinase with disintegrin and thrombospondin domains
ARF6: ADP-ribosylation factor 6
BCRP: Breast cancer resistance protein
CSF: Cerebrospinal fluid
ECM: Extracellular matrix
EGFR: Epidermal growth factor receptor
EMMPRIN: Extracellular matrix metalloproteinase inducer
EMT: Endothelial to mesenchymal transition
ERK: Extracellular signal-regulated kinase
EV: Extracellular vesicle
FAK: Focal adhesion kinase
FN: Fibronectin
HGF: Hepatocyte growth factor
LIMK: LIM kinase
MDR: Multidrug resistance
MLCK: Myosin light-chain kinase
MMP: Matrix metalloproteinase
MRP1: Multidrug resistance-associated protein 1
PAD: Peptidylarginine deiminase
PE: Phosphatidylethanolamine
PMV: Platelet-derived microvesicle
PS: Phosphatidylserine
PSGL: P-selectin glycoprotein ligand
ROCK: Rho-associated coiled coil containing protein kinase
ROS: Reactive oxygen species
TF: Tissue factor
TGF-β: Transforming growth factor β
TNF-α: Tumor necrosis factor α
TMV: Tumor-derived microvesicle
tTG: Tissue transglutaminase
uPA: Urokinase plasminogen activator
VEGF: Vascular epithelium growth factor
